# Figure Disembedding: The Gottschaldt’s Hidden Figure Test in Children with Typical Development and Autism

**DOI:** 10.1007/s10803-021-05259-3

**Published:** 2021-09-02

**Authors:** Massimiliano Conson, Mattia Siciliano, Luigi Trojano, Pierluigi Zoccolotti, Isa Zappullo, Chiara Baiano, Giovanni Caputo, Alessandro Russo, the LabNPEE group, Roberta Cecere, Alessandro Di Rosa, Giovanna Esposito, Rosa Milo, Francesco Polito, Barbara Rauso, Maria Vela, Gabriella Santangelo

**Affiliations:** 1 Department of Psychology, University of Campania Luigi Vanvitelli, Caserta, Italy; 2 Department of Advanced Medical and Surgical Sciences, University of Campania Luigi Vanvitelli, Naples, Italy; 3 Department of Psychology, Sapienza University of Rome, Rome, Italy; 4 Institute for Cognitive Sciences and Technologies (ISTC–CNR), Rome, Italy; 5 Center for Studies and Research “Caputo & Ippolito – Multisystemic Aquatic Therapy”, Casoria, Naples, Italy; 6 Laboratory of Developmental Neuropsychology, Department of Psychology, University of Campania Luigi Vanvitelli, Viale Ellittico 31, 81100 Caserta, Italy

**Keywords:** Figure disembedding, Hidden figures, Perceptual style, Perception of details

## Abstract

In two studies, we used the Gottschaldt’s Hidden Figure Test (GHFT) for assessing figure disembedding ability in children aged 7–11. Study 1 demonstrated in a large sample of typically developing children that GHFT accuracy and time scores differed across age groups, without sex and socioeconomic differences. Thus, we provided normative data only taking into account children’s age. In Study 2, GHFT normative values were used to assess children with autism, who were also compared with a closely age-matched group of typical controls. Children with autism achieved time scores at or above the 50th centile and significantly differed from the controls for time score. The GHFT seems a valuable tool for defining the cognitive profile of children with autism.

## Introduction

Figure disembedding ability is the capacity to visually locate and detect local elements immersed within a global configural shape (Witkin, 1950; Witkin et al., 1971). It has been classically demonstrated that, when participants are required to search for a simple figure (local level) integrated in a larger one (global level), their task is more difficult if the lines of the simple figure belong perceptually to a different visual configuration within the complex figure, an effect early referred to as “embeddedness” by Gottschaldt (1926, 1929).

Gottschaldt (1926, 1929) introduced the Embedded Figure Test as a suitable measure of the ability to disentangle a figure from the background. The test material is a series of meaningless geometrical patterns in which a simpler geometrical figure is embedded, and the task requirement is to pencil it in. Following the original Gottschaldt’s Hidden Figure Test (GHFT), different versions of the Embedded Figures test have been devised, in which individuals are required to identify a target (simple) shape within complex designs. The most known version is that developed by Witkin et al. (1971).

The ability of disembedding figures undergoes developmental changes across childhood, with younger children being less able to detect embedded figures from the background, as indexed by both time and accuracy measures (Amador-Campos & Kirchner-Nebot, 1997; Cecchini & Pizzamiglio, 1975; Goodenough & Eagle, 1963; Witkin et al., 1967). Moreover, children’s performance seems to be affected by socioeconomic status and sex, with better scores associated to higher socioeconomic status and to male sex, although the results are not entirely consistent (Cakan, 2003; Cecchini & Pizzamiglio, 1975; Forns-Santacana et al., 1993; Karp et al., 1969; Witkin et al., 1967).

Persons with autism are thought to perform better than typical controls in disembedding figures. In a seminal study, Shah and Frith (1983) observed superior performance of children with autism on the Children’s Hidden Figure Test (Witkin et al., 1971) and explained this finding in terms of an enhanced ability to focus on the details within the whole, ignoring the interfering effect of the overall configuration (gestalt). Other studies reported that individuals with autism are as accurate as typical controls in disembedding figures but often show faster response times (Horlin et al., 2016). The inconsistencies in the literature are probably due to methodological reasons such as heterogeneity of task versions, stimuli presentation, and response recording (for a discussion see White & Saldaña, 2011).

To account for the advantage of individuals with autism in disembedding figures, the “Weak Central Coherence” (WCC) model proposed that the perceptual profile in these individuals is characterized by a weakness in global processing of information and a tendency towards local processing (Frith, 1989). Such local processing style would not be intrinsically disadvantageous, but rather it would be related to the specific requirements of the task at hand, for instance being advantageous in tasks requiring a superior ability to process details, such as the Figure Disembedding and the Block Design tests (Happé & Frith, 2006). From another angle, the “Enhanced Perceptual Functioning” (EPF) model posited that individuals with autism would show an enhanced capacity in local processing rather than an impaired ability for global processing (Mottron & Burack, 2001; Mottron et al., 2006).

To address the complex differences between children with typical development and children with autism in figure disembedding ability in the clinical setting, we deemed useful developing a standardized version of the GHFT, with robust normative data available for both time and accuracy parameters. Hence, in the present research, we: (1) assessed developmental changes across childhood in GHFT performance; (2) provided normative data from a large sample of native Italian speaking children; (3) tested a group of children with autism on the standardized version of the GHFT.

To these aims, two studies were conducted. In both, we adopted the GHFT version used by Capitani et al. (1988), in which participants have to complete a first series of stimuli within 15 min and a second series within 5 min. As we assess children, we did not impose any time limit as in adult individuals, but rather recorded the time (s) needed to complete the whole series of patterns.

Study 1 assessed the changes in performance on the GHFT as a function of age and education in a sample of 7–11-year-old typically developing children. This age range was chosen since age consistently affects figure disembedding ability (Amador-Campos & Kirchner-Nebot, 1997; Bigelow, 1971; Cecchini & Pizzamiglio, 1975; Witkin et al., 1967), with a progressive maturation of global and local perceptual abilities, particularly during the early school period (Dukette & Stiles, 2001; Kimchi et al., 2005; Poirel et al., 2008). Response time and accuracy scores of the GHFT were computed using the LMS method (Cole & Green, 1992), which allowed for obtaining normalized growth centile standards.

Study 2 evaluated the GHFT performance of children with autism with respect to the normative data gathered from participants in Study 1, and also compared performance of the autism group with that of closely age-matched typically developing controls.

## Methods

**Fig. 1:**
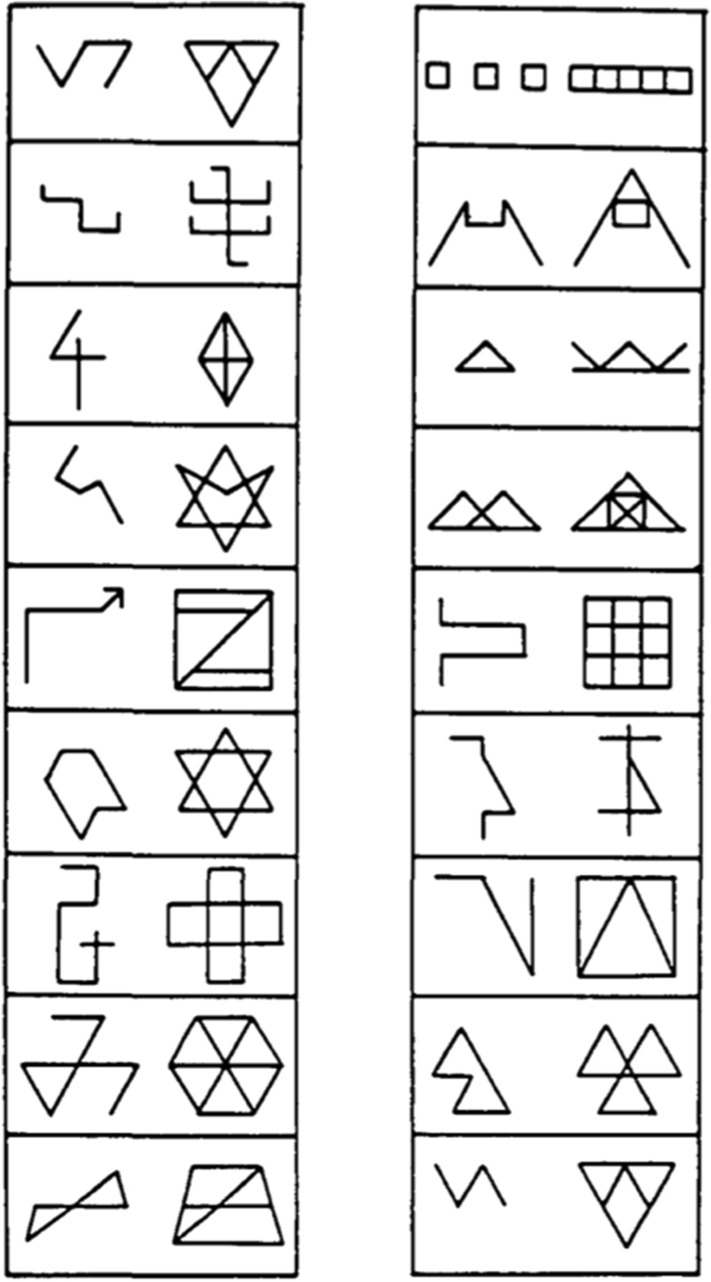
Example of the stimuli from Tables 1 and 2 of the GHFT

In the GHFT, participants are presented with 34 complex geometrical figures in which a simple shape is hidden (Capitani et al., 1988; some examples are provided in Fig. 1). Test stimuli are arranged in four tables. In the first three tables containing nine items each, participants need to search for the simple figure (on the left side) within the complex figure (on the right side) and highlight it in by a pencil. In the last table, instead, the simple figure is located at the center of the sheet; the participants have to identify it within seven different complex figures placed above and below the simple shape. Each correct choice is scored 1 (score range 0–34) to obtain the accuracy score. The time needed to solve each of the four tables is recorded and their sum provides the total time score.

The study was conducted according to the standards of the Helsinki Declaration and the study protocol was approved by the local ethical committee of the Department of Psychology of the University of Campania Luigi Vanvitelli (code: N:34/03.11.2020). Written informed consent was obtained from the parents of each participant involved in the study.

### Study 1

#### Participants

Typically developing children were recruited from elementary schools located in the Campania region, Southern Italy. To be included in the study, each participant had to meet the following inclusion criteria: (i) a normal score (≥ 15th percentile of the Italian normative data; Pruneti et al., 1996) at the Raven’s Colored Progressive Matrices test (RPM; Raven et al., 1998); (ii) age range from 7 to 11 years; (iii) lack of neurologic, neuropsychological, or neuropsychiatric disorders, as reported by either parents or teachers; and (iv) Italian as native language. We recruited a sample of 403 children (188 males).

Children’s socioeconomic status (SES) was measured using the Hollingshead Four Factor Index of social status (Hollingshead, 1975), which estimates SES based on a weighted average of education and occupational level of both parents of each child (Venuti & Senese, 2007).

#### Statistical Analysis

An a priori power analysis for one-way analysis of variance (ANOVA) was carried-out by G*Power (Version 3.1.9.2), setting the following parameters: probability level (α), 0.05; statistical power (1 − β), 0.80; moderate effect size (Cohen’s f of 0.25) (Cohen, 1988).

The overall sample was divided into eight groups (Table 1); as in previous normative studies (e.g., Conson et al., 2019; Mozzanica et al., 2016), each group covered a 6-month range.

**Table 1:** Normative sample stratified by age

Age range _(years.months–years.months)_	Total
7.0–7.5	34
7.6–7.11	56
8.0–8.5	49
8.6–8.11	63
9.0–9.5	51
9.6–9.11	60
10.0–10.5	56
10.6–10.11	34
Total	403

SES was split into two groups (i.e., high and low SES) using the K-means clustering procedure (non-hierarchical clustering) since no cut-off value is available in the literature.

Three one-way ANOVAs were carried out for evaluating the effect of age groups, sex, and SES on time and accuracy scores of the GHFT. Tukey’s honestly significant differences (HSD) tests were used for post-hoc comparisons.

Centiles for time and accuracy scores of the GHFT were computed using the LMS method (Cole & Green, 1992), which allows for obtaining normalized growth centile standards. The method assumes that data can be normalized using a power function, which stretches one tail of the distribution while shrinking the other. The optimal power (i.e., Box–Cox power transformation) to obtain normality was calculated for each age group and the trend summarized by a smooth (L) curve. Trends in the mean (M) and coefficient of variation (S) were similarly smoothed.

The resulting L, M, and S curves contain the information to draw any centile by the following formula:

$${\text{C }} = {\text{ M}}\left( {{1} + {\text{LSZ}}} \right){1}/{\text{L}}$$

where Z is the value of the z-score corresponding to centile. The 3rd, 5th, 10th, 15th, 25th, 50th, 75th, 90th, and 97th centiles were chosen as age-specific reference values.

All analyses were performed using IBM Statistical Package for Social Science (SPSS; Version 21; IBM Corp., Armonk, NY, USA), with p-value < 0.05 considered as statistically significant.

#### Results

The a priori power analysis revealed that at least 240 participants (i.e., 30 individuals for each age group) were needed to attain a moderate effect size.

K-means clustering identified two clusters with high (M = 44.73, SD = 9.07) and low (M = 24.29, SD = 7.97) SES score, containing 191 and 212 participants, respectively. Descriptive statistics for each age group are shown in Table 2.

**Table 2:** Descriptive statistics stratified by age range are shown as mean (standard deviation) or count (percentage), as appropriate

				Age range _(years.months – years.months)_					
	7.0–7.5	7.6–7.11	8.0–8.5	8.6–8.11	9.0–9.5	9.6–9.11	10.0–10.5	10.6–10.11	Total
Age, months	87.11 (1.66)	92.60 (1.81)	98.69 (1.84)	104.50 (1.87)	110.62 (1.55)	116.75 (1.62)	122.58 (1.77)	128.47 (1.76)	107.81 (12.75)
Education, years	2.03 (0.17)	2.17 (0.38)	2.63 (0.48)	3.11 (0.31)	3.72 (0.45)	4.23 (0.53)	4.82 (0.38)	5.00 (0.00)	3.47 (1.10)
Sex, male	11 (32%)	23 (41%)	24 (49%)	33 (52%)	25 (49%)	30 (50%)	27 (48%)	15 (44%)	188 (47%)
SES	32.00 (18.20)	36.33 (16.86)	38.59 (15.64)	32.25 (16.96)	29.50 (16.66)	32.72 (12.01)	34.35 (14.79)	32.98 (15.27)	33.99 (15.72)
CPM	22.44 (4.76)	23.39 (4.33)	26.36 (3.94)	26.47 (3.57)	28.00 (3.76)	28.58 (3.72)	29.75 (3.46)	30.67 (2.92)	27.00 (4.55)
*GHFT:*									
Accuracy	16.97 (7.84)	18.80 (12.14)	21.77 (6.83)	23.69 (5.70)	24.09 (6.32)	25.28 (4.50)	27.78 (4.47)	25.70 (5.58)	23.24 (7.71)
Time (s)	592.85 (211.43)	573.21 (267.46)	547.18 (189.34)	537.31 (147.14)	511.41 (192.63)	488.73 (211.21)	476.87 (151.95)	445.14 (132.79)	521.50 (196.75)

*SES* socioeconomic status; *CPM* coloured progressive matrices, *GHFT* Gottschaldt's Hidden Figure Test

One-way ANOVAs showed a significant effect of age group on time (F = 2.85, p < 0.01, η^2^_p_ = 0.05), and accuracy (F = 12.03, p < 0.01, η^2^_p_ = 0.18) scores, but not of sex and SES (p > 0.05).

Tukey HSD post-hoc comparisons showed statistically significant differences in time scores between the 7.0–7.5 and 10.6–10.11 age groups, and a marginally significant difference between the 7.6–7.11 and 10.6–10.11 age groups. As for accuracy score, statistically significant differences were found between the 7.0–7.5 or the 7.6–7.11 and the 8.6–8.11, 9.0–9.5, 9.6–9.11, 10.0–10.5, or 10.6–10.11 age groups. Similarly, statistically significant differences were found between 8.0–8.5 or 8.6–8.11 and 10.0–10.5 age groups (Table 3).

**Table 3:** Tukey’s honestly significant differences (HSD) post-hoc test results for time (above the diagonal) and accuracy (below the diagonal) of Gottschaldt's Hidden Figure Test

Age range _(years,months–years,months)_	7.0–7.5	7.6–7.11	8.0–8.5	8.6–8.11	9.0–9.5	9.6–9.11	10.0–10.5	10.6–10.11
7.0–7.5		1.00	0.96	0.88	0.55	0.19	0.11	*0.03*
7.6–7.11	0.93		0.99	0.97	0.72	0.27	0.14	0.05
8.0–8.5	0.05	0.38		1.00	0.98	0.76	0.58	0.26
8.6–8.11	< *0.01*	< *0.01*	0.84		0.99	0.86	0.68	0.33
9.0–9.5	< *0.01*	< *0.01*	0.72	1.00		0.99	0.98	0.78
9.6–9.11	< *0.01*	< *0.01*	0.17	0.92	0.98		1.00	0.96
10.0–10.5	< *0.01*	< *0.01*	< *0.01*	*0.04*	0.13	0.55		0.99
10.6–10.11	< *0.01*	< *0.01*	0.20	0.88	0.97	1.00	0.88	

Significant differences are signed in italic

**Fig. 2:**
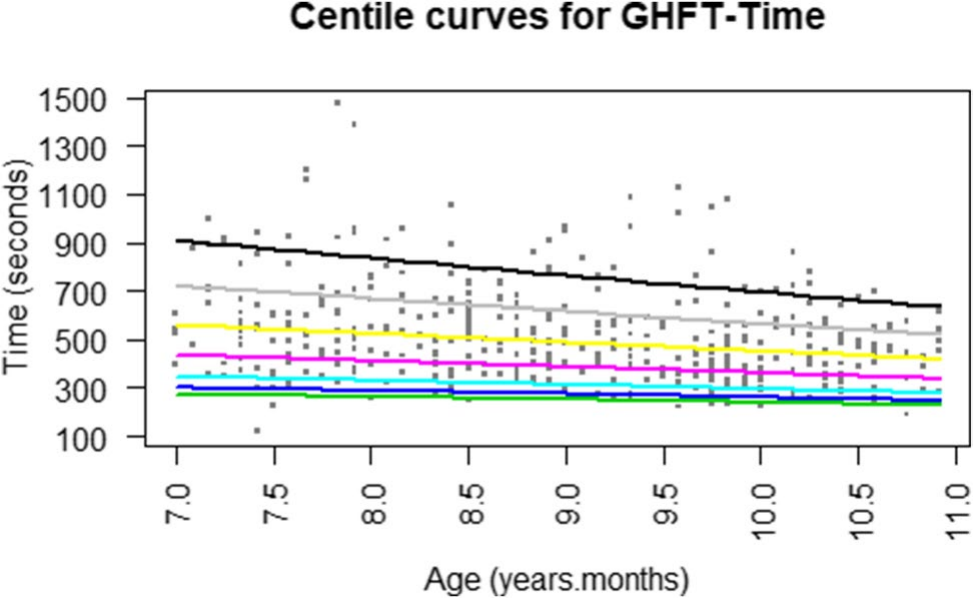
Estimates of GHFT time centiles. Green, blue, light blue, purple, yellow, grey, and black curves represent the 3rd, 5th, 10th, 25th, 50th, 75th, and 90th percentiles, respectively (Color figure online)

**Fig. 3:**
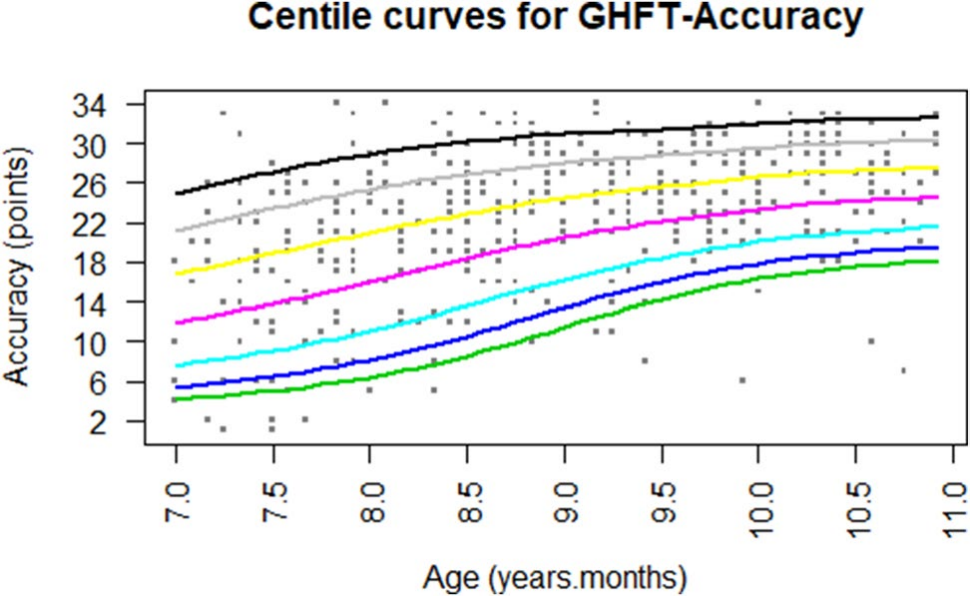
Estimate of GHFT accuracy centiles. Green, blue, light blue, purple, yellow, grey, and black curves represent the 3rd, 5th, 10th, 25th, 50th, 75th, and 90th percentiles, respectively (Color figure online)

Centile curves (Cole & Green, 1992) for time and accuracy scores are provided in Figs. 2 and 3, respectively.

Time and accuracy centiles are reported in Table 4. Note that, when the percentile of interest is not available, it is possible to compute it by the formula reported above and considering the LMS parameters associated with each age group (Table 4). For example, to compute the 95th percentile (corresponding to a z score equal to 1.64) of the accuracy score for the 9.0–9.5 age group, the formula becomes 24 × (1 + 1.35 × 0.27 × 1.64)1/1.35 = 33.94 which can be approximated to integer score of 34.

**Table 4:** Age-specific percentiles for time and accuracy of Gottschaldt's Hidden Figure Test

Age range(years, months–years, months)	L	S	Centiles								
			3rd	5th	10th	15th	25th	50th (M)	75th	90th	97th
Time (s)											
7.0–7.5	0.73	0.37	1034	973	883	822	736	583	439	320	213
7.6–7.11	− 0.48	0.39	1295	1125	920	812	684	514	399	326	272
8.0–8.5	− 0.02	0.34	989	911	803	737	651	516	410	333	272
8.6–8.11	0.31	0.27	850	803	735	690	628	524	431	358	295
9.0–9.5	− 0.30	0.34	979	885	762	692	602	472	376	310	259
9.6–9.11	− 0.53	0.37	1050	917	757	672	571	436	344	284	240
10.0–10.5	0.17	0.32	816	761	683	634	567	457	366	297	240
10.6–10.11	0.97	0.31	708	675	624	589	539	445	351	267	185
Accuracy											
7.0–7.5	0.86	0.55	1	3	6	7	11	17	23	29	34
7.6–7.11	0.87	0.45	4	5	8	10	13	18	24	29	34
8.0–8.5	1.22	0.33	7	9	12	14	17	22	27	31	34
8.6–8.11	1.30	0.24	12	13	16	17	20	24	28	31	34
9.0–9.5	1.35	0.27	10	12	15	17	20	24	29	32	34
9.6–9.11	1.31	0.17	16	17	19	20	22	25	28	31	33
10.0–10.5	2.59	0.15	16	19	21	23	25	28	31	33	34
10.6–10.11	1.96	0.20	13	15	18	20	22	26	29	32	34

### Study 2

#### Participants

Twenty-two individuals with autism (three female; mean age = 9.2, SD = 1.43; age range 7–11) took part in the study. To be included in the study, each participant had to meet the same inclusion criteria as in Study 1.

Diagnosis of autism was reached after a multidisciplinary assessment by a neuropsychiatrist and a clinical psychologist trained in the evaluation of individuals with neurobehavioural disorders according to DSM-V criteria. Clinical diagnosis was validated by means of the Autism Diagnostic Interview-Revised (ADI-R; Rutter et al., 2003) and the Autism Diagnostic Observation Schedule Module 3 (ADOS-2; Lord et al., 2012).

We also recruited a sample of 22 typically developing children (three females; mean age = 9.2, SD = 1.44; age range 7–11) individually matched for age and sex with autistic children. Typically developing children were recruited from primary schools in Naples, in the Campania Region of Italy.

Since the RPM scores are well correlated with Wechsler Full Scale intelligence quotient (IQ) (e.g., O’Leary et al., 1991), they were used to estimate IQ. The estimated IQ of children with typical development (mean = 101.2, SD = 11.6) did not significantly differ (t-test = − 1.49, p = 0.143) from that of children with autism (mean = 106.3, SD = 11.2).

#### Statistical Analysis

A priori power analyses for sample size calculation were conducted with G*Power 3.1 by setting the following parameters: probability level (α) of 0.05, statistical power (1 − β) of 0.80, and large effect size (Cohen’s d of 0.80 for t-test and odds ratio of 6.71 for binary multiple logistic regression analysis) (Cohen, 1988; Faul et al., 2009).

We first compared autistic children’s performance with respect to the normative data gathered in Study 1.

Then, independent t-tests were conducted on both response time and accuracy scores of the GHFT as a function of groups (autistic children vs typical controls).

Finally, a binary multiple logistic regression analysis was performed to identify the scores of GHFT which were able to discriminate the children with autism from typically developing controls. To check for the reliability of the results due to the relatively small sample size, we computed 95% bias corrected and accelerated confidence intervals [95% CI] (1000 bootstrap samples) for the logistic regression coefficients. The bias of an estimate can be ignored if it is lower than 0.25 times its standard error.

The analysis was performed using IBM Statistical Package for Social Science (SPSS; Version 21), with p value < 0.05 considered as statistically significant.

#### Results

**Fig. 4:**
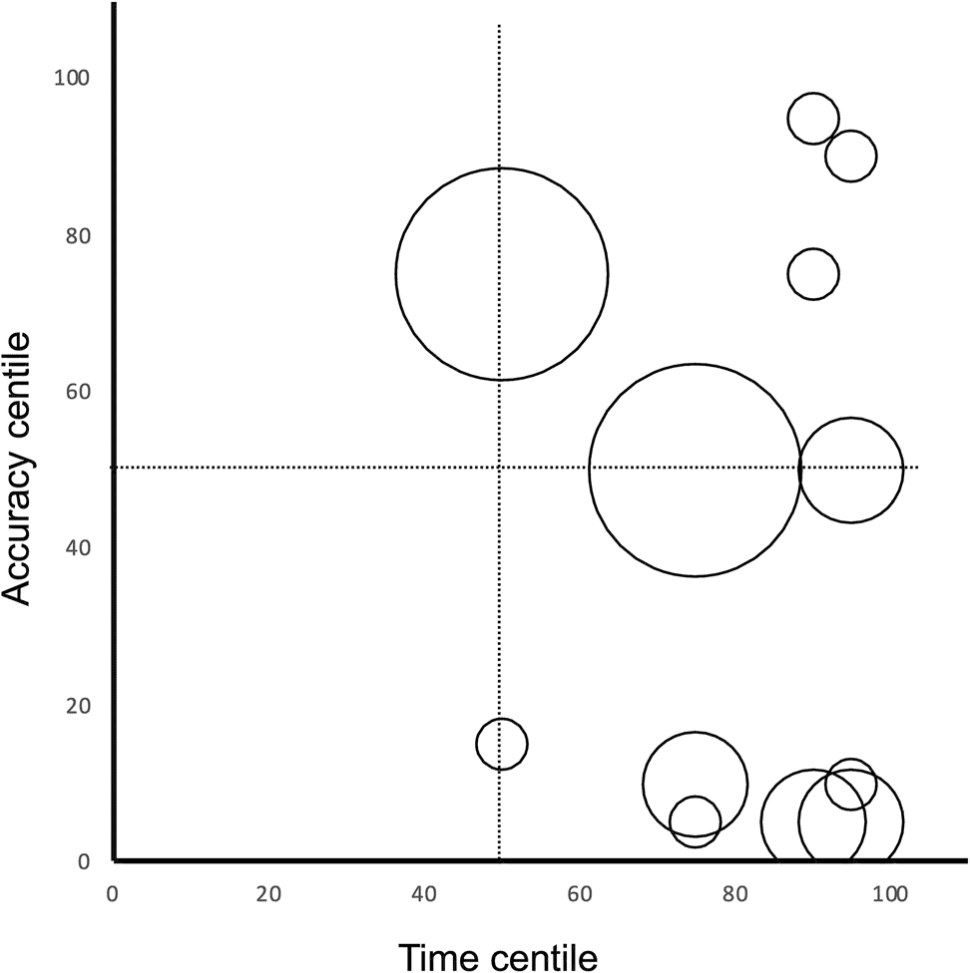
Percentiles of age-corrected GHFT accuracy and time scores achieved by single individuals with autism in reference to normative data obtained from Study 1. Dimension of symbol is proportional to density of observations (the smallest symbols represent one individual, the largest four individuals). The dotted lines represent the 50th percentile

With reference to the normative data obtained from the Study 1, all children with autism had a time score at or above the 50% centile of the normative data, and particularly six of them (27.3% of the sample) had a time score above the 90th centile. By contrast, their accuracy scores ranged across the whole percentile range (Fig. 4).

As it regards the comparison between children with autism with the age-matched controls, the a priori power analyses revealed that at least 42 participants (21 for each group) for t-test and 34 participants for binary multiple logistic regression analysis were needed to attain a large effect size, at a statistical power of 0.80 and α level of 0.05.

**Fig. 5:**
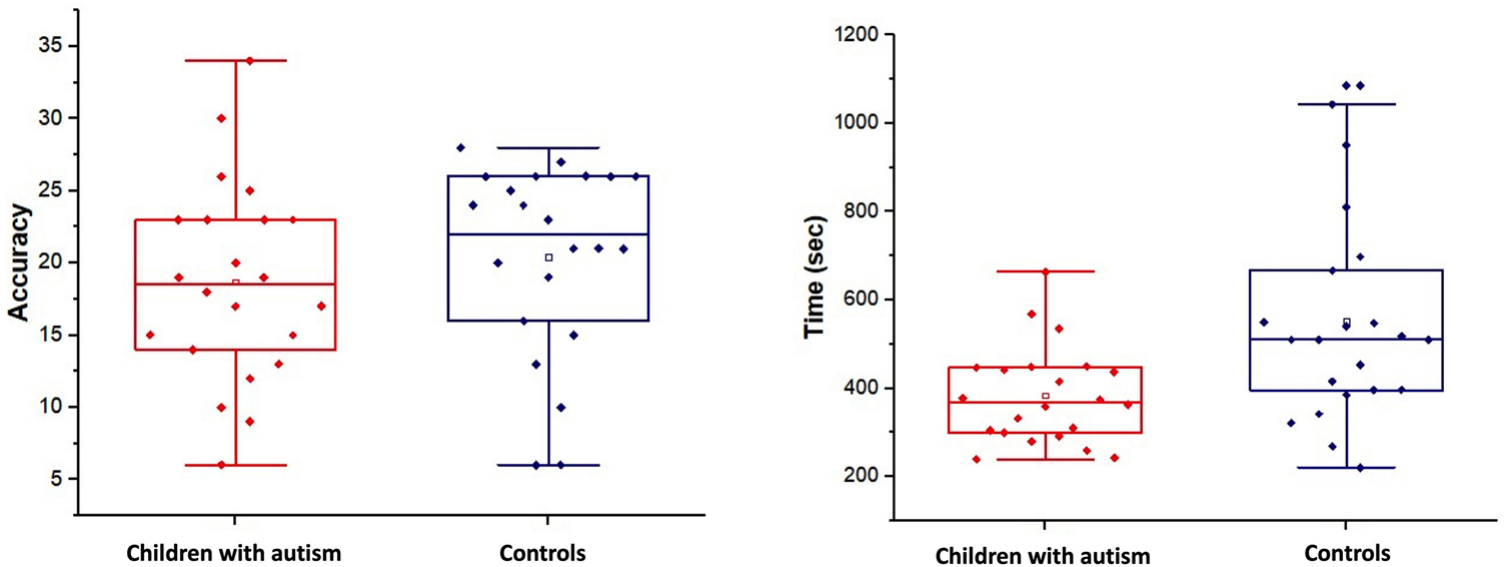
Time and accuracy scores of the GHFT, separately for children with autism and typically developing participants. Boxes represent 25 and 75 percentiles. The solid line inside the box represents the median of the group, while the empty square in the box represents the mean. Bars above and below the boxes represent the interquartile range. Each individual dot represents a subject

Mean response time and accuracy scores of the GHFT are reported in Fig. 5 separately for each group. Results of independent t-tests showed that children with autism were significantly faster (t-test = 3.01, p = 0.004, Cohen’s d = 0.90) than typically developing controls, while no significant difference was found on the total accuracy score (t-test = − 0.84, p = 0.40, Cohen’s d = 0.26).

The results of the binary multiple logistic regression analysis showed that only the time score of GHFT discriminated children with autism from typically developing controls (p = 0.01) with an overall accuracy of 68.2%. The bias estimates of the regression coefficients were lower than 0.25 times their standard errors, indicating no substantial bias, and thus, adequacy of the sample size (Table 5).

**Table 5:** Results of the binary multiple logistic regression analysis

Variable	Bias	Beta (SE)	Wald	*p*-value	OR [95% CI]
GHFT-time score	− 0.00	− 0.00 (0.00)	5.69	**0.01**	0.99 [0.98, 0.99]
GHFT-accuracy score	− 0.00	− 0.03 (0.06)	0.40	0.52	0.96 [0.88, 1.06]

Model χ2(2) = 9.84, *p* < 0.01, R^2^(Nagelkerke) = 0.27; variables which were able to discriminate children with autism from typically developing controls were shown in bold

## Discussion

The main aims of the present investigation were, first, assessing developmental changes across childhood in GHFT performance and providing normative data on Italian language speaking children (Study 1), and second, assessing a group of children with autism on the standardized version of the GHFT (Study 2).

Results of Study 1 showed that, although a progressive reduction of time scores could be appreciated across all the age ranges, only the two farther age groups were significantly different. These results are in line with Witkin et al.’s (1967) data showing an overall progressive tendency for children to become faster at disembedding, with a levelling off of the trend from about 14 years onward. The accuracy score was statistically lower in 7.0–7.5 and 7.6–7.11 groups with respect to all the other age groups, and in 8.0–8.5 or 8.6–8.11 groups with respect to 10.0–10.5 age groups, thus suggesting a smooth increase of accuracy across childhood. These findings are consistent with previous data on elementary school children (Amador-Campos & Kirchner-Nebot, 1997), with a particular refinement of performance when comparing 7-year-old children with the older ones (Bigelow, 1971; Cecchini & Pizzamiglio, 1975).

Here we did not detect differences due to sex or socioeconomic status. Thus, the normative data only took into account the effect of age. Sex differences have been reported in the literature on figure disembedding but with some inconsistencies partially accounted for by age. In age ranges well comparable with the present one, significant sex differences did not emerge (Bigelow, 1971; Cecchini & Pizzamiglio, 1975; Corah, 1965) although a non-significant advantage of boys over girls was also reported (Amador-Campos & Kirchner-Nebot, 1997). Indeed, until age 10, boys and girls display similar figure disembedding abilities while they tend to diverge during adolescence (Witkin et al., 1967). As for the possible effect of SES on performance, in a review of studies on children aged 4.5–10.5 years Laicardi-Pizzamiglio and Pizzamiglio (1974) found that poorer figure disembedding was related to lower socioeconomic status, but this finding could be related to participant’s general cognitive abilities (Bigelow, 1971; Forns-Santacana et al., 1993). It is possible that in studies on children with a narrow range of general cognitive functioning the differences related to socioeconomic status in figure disembedding could be weakened, consistent with recent findings (Zappullo et al., 2020).

Results of Study 2 showed that children with autism were significantly faster than typical controls whereas the two groups did not differ in accuracy. This finding was also clear with reference to the normative data in Study 1: a substantial proportion of autistic children achieved a time score in the highest centiles of the normative distribution, but only in some of them the accuracy score was at high centiles.

It has been suggested that methodological factors affecting task performance at a behavioral level, rather than a true difference between children with autism and typical development in figure disembedding at a cognitive level, could account for reported group differences on figure disembedding tests (White & Saldaña, 2011). Indeed, several approaches in the analysis of response time differences between children with autism and typical development are available in literature each producing somewhat different data. Some authors computed the average response times for only correct responses (e.g., de Jonge et al., 2006; Morgan et al., 2003), whilst others examined response times to all stimuli, either replacing the maximum time allowed for incorrect trials (e.g., Jolliffe & Baron-Cohen, 1997; Ropar & Mitchell, 2001) or including searching times regardless of whether the response was correct or not (Edgin & Pennington, 2005). Here, we followed the last procedure and recorded the time (in s) needed to complete the whole series of 34 items, irrespectively of the correctness of the responses. The present results were consistent with Edgin and Pennington (2005) and supported the strength of children with autism in disembedding. White and Saldaña (2011) suggested that considering time irrespective of trial accuracy could be affected by different strategies used to detect targets on incorrect trials, as some participants might give up search for the target more quickly than others. However, White and Saldaña’s observations (2011) mainly referred to Witkin’s task or to comparable versions, as the Coates’ (1972) one, in which, for each trial, the participant has to locate a simple target figure (e.g., a triangle) within a complex picture. Such task versions can favor a practice effect, since one or two target figures have to be repeatedly searched within complex images (Gottschaldt, 1926; Ludwig & Lachnit, 2004; Witkin et al., 1967). Moreover, in the Witkin’s task, a small number of items is presented (12 items), thus often leading to a ceiling effect (De Jonge et al., 2006; Jolliffe & Baron-Cohen, 1997). Practice in disembedding could reflect the impact of experience in terms of both perceptual and procedural (searching strategy) factors but can be reduced by varying the stimulus material as in the GHFT (Ludwig & Lachnit, 2004).

In the GHFT, 27 pairs of different images are provided in Tables 1, 2 and 3, whereas in the last table only one target has to be identified within seven different complex images. Hence, this set-up allowed to markedly increase task complexity with respect to other available versions, such as Witkin’s or Coates’ tests, counteracting the impact of practicing. Indeed, we did not find evidence of ceiling in any of the tested populations. Importantly, increased task complexity, and reduced practice effect, are thought to affect response time more than accuracy (Ludwig & Lachnit, 2004; Zoccolotti & Pizzamiglio, 1982), thus favoring the sensitivity of response times in discriminating between participants with autism and typically developing controls. Our results from the comparison of autistic children’s performance with both normative data and individually matched controls consistently showed that GHFT faster time scores significantly relate to the presence of autism, consistent with evidence demonstrating that individuals with autism are able to locate the embedded figures more quickly than controls (de Jonge et al., 2006; Jarrold et al., 2005; Jolliffe & Baron-Cohen, 1997; Morgan et al., 2003; Pellicano et al., 2005).

Both the WCC (Happé & Frith, 2006) and the EPF (Mottron et al., 2006) predict superior disembedding abilities in individuals with autism, albeit hypothesizing different underpinning mechanisms. Comparing the explanatory power of these two models would require more complex paradigms than the one adopted in the present investigation (Horlin et al., 2016). Thus, our findings do not allow to shed light on the reasons why children with autism are faster than typically developing controls in disembedding figures. Moreover, we have to mention that a further limitation of the present study is the reduced possibility to generalize the results to age ranges different from that involved here. Indeed, as recalled above, critical developmental changes occur in global and local perception abilities during the elementary school period, but a progressive refinement of these abilities protracts across adolescence (Mondloch et al., 2003). Hence, future studies should test the GHFT in typically developing adolescents and assess its validity to test figure disembedding abilities in adolescents with autism.

In conclusion, the results of Study 1 on a large sample of typically developing children aged 7–11 demonstrated that GHFT accuracy and time scores differed across age groups, without sex and socioeconomic differences. Thus, we could provide normative data only considering children’s age. In Study 2, children with autism achieved time scores at or above the 50th centile with respect to normative values and significantly differed for time scores from a closely age-matched group of typically developing controls. Taken together, these findings indicate that the GHFT is a valuable tool for assessing developmental changes in children’s figure disembedding ability and tracing the functional cognitive profile of children with autism.

## References

[CR1] Amador-Campos JA, Kirchner-Nebot T (1997). Relations of scores on Children's Embedded Figures Test with age, item difficulty and internal consistency. Perceptual and Motor Skills.

[CR2] Bigelow GS (1971). Field dependence-field independence in 5- to 10-year-old children. The Journal of Educational Research.

[CR3] Cakan M (2003). Cross-cultural aspect of the group embedded figures test: Norms for Turkish eighth graders. Perceptual and Motor Skills.

[CR53] Capitani E, Sala SD, Lucchelli F, Soave P, Spinnler H (1988). Perceptual attention in aging and dementia measured by Gottschaldt’s hidden figure test. Journal of Gerontology.

[CR4] Cecchini M, Pizzamiglio L (1975). Effects of field-dependency, social class and sex of children between ages 5 and 10. Perceptual and Motor Skills.

[CR5] Coates SW (1972). Preschool embedded figures test.

[CR6] Cohen J (1988). Statistical power analysis for the behavioral sciences.

[CR7] Cole TJ, Green PJ (1992). Smoothing reference centile curves: The LMS method and penalized likelihood. Statistics in Medicine.

[CR8] Conson M, Siciliano M, Baiano C, Zappullo I, Senese VP, Santangelo G (2019). Normative data of the Rey–Osterrieth Complex Figure for Italian-speaking elementary school children. Neurological Sciences.

[CR9] Corah NL (1965). Differentiation in children and their parents. Journal of Personality.

[CR10] de Jonge MV, Kemner C, van Engeland H (2006). Superior disembedding performance of high-functioning individuals with autism spectrum disorders and their parents: The need for subtle measures. Journal of Autism and Developmental Disorders.

[CR11] Dukette D, Stiles J (2001). The effects of stimulus density on children’s analysis of hierarchical patterns. Developmental Science.

[CR12] Edgin JO, Pennington BF (2005). Spatial cognition in autism spectrum disorders: Superior, impaired, or just intact?. Journal of Autism and Developmental Disorders.

[CR13] Faul F, Erdfelder E, Buchner A, Lang AG (2009). Statistical power analyses using G*Power 3.1: Tests for correlation and regression analyses. Behavior Research Methods.

[CR14] Forns-Santacana M, Amador-Campos JA, Roig-Lopez F (1993). Differences in field dependence-independence cognitive style as a function of socioeconomic status, sex, and cognitive competence. Psychology in the Schools.

[CR15] Frith U (1989). Autism: Explaining the enigma.

[CR16] Goodenough DR, Eagle CJ (1963). A modification of the embedded-figures test for use with young children. Journal of Genetic Psychology.

[CR17] Gottschaldt K (1926). Üeber den Einfluss der Erfahrung auf die Wahrnehmung von Figuren, I. Psychologische Forschung.

[CR18] Gottschaldt K (1929). Üeber den Einfluss der Erfahrung auf die Wahrnehmung von Figuren, II. Psychologische Forschung.

[CR20] Happé F, Frith U (2006). The weak coherence account: Detail-focused cognitive style in autism spectrum disorders. Journal of Autism and Developmental Disorders.

[CR21] Hollingshead A (1975). The four-factor index of social status.

[CR22] Horlin C, Black M, Falkmer M, Falkmer T (2016). Proficiency of individuals with autism spectrum disorder at disembedding figures: A systematic review. Developmental Neurorehabilitation.

[CR23] Jarrold C, Gilchrist ID, Bender A (2005). Embedded figures detection in autism and typical development: Preliminary evidence of a double dissociation in relationships with visual search. Developmental Science.

[CR24] Jolliffe T, Baron-Cohen S (1997). Are people with autism and Asperger syndrome faster than normal on the Embedded Figures Test?. Journal of Child Psychology and Psychiatry, and Allied Disciplines.

[CR25] Karp SA, Silberman L, Winters S (1969). Psychological differentiation and socioeconomic status. Perceptual and Motor Skills.

[CR27] Kimchi R, Hadad B, Behrmann M, Palmer SE (2005). Microgenesis and ontogenesis of perceptual organization: Evidence from global and local processing of hierarchical patterns. Psychological Science.

[CR29] Laicardi-Pizzamiglio C, Pizzamiglio L (1974). Psychometric data of some tests of field dependence related to an Italian population of children from 4% to 10% years. Archivio De Psicologia Neurologica.

[CR30] Lord C, Rutter M, DiLavore PC, Risi S, Gotham K, Bishop SL (2012). Autism diagnostic observation schedule, (ADOS-2), Part 1: Modules 1–4.

[CR31] Ludwig I, Lachnit H (2004). Effects of practice and transfer in the detection of embedded figures. Psychological Research Psychologische Forschung.

[CR32] Mondloch CJ, Geldart S, Maurer D, de Schonen S (2003). Developmental changes in the processing of hierarchical shapes continue into adolescence. Journal of Experimental Child Psychology.

[CR33] Morgan B, Maybery M, Durkin K (2003). Weak central coherence, poor joint attention, and low verbal ability: Independent deficits in early autism. Developmental Psychology.

[CR34] Mottron L, Burack J, Burack JA, Charman T, Yirmiya N, Zelazo PR (2001). Enhanced perceptual functioning in the development of autism. The development of autism: Perspectives from theory and research.

[CR35] Mottron L, Dawson M, Soulières I, Hubert B, Burack J (2006). Enhanced perceptual functioning in autism: An update, and eight principles of autistic perception. Journal of Autism and Developmental Disorders.

[CR36] Mozzanica F, Salvadorini R, Sai E, Pozzoli R, Maruzzi P, Scarponi L, Barillari MR, Spada E, Ambrogi F, Schindler A (2016). Reliability, validity and normative data of the Italian version of the bus story test. International Journal of Pediatric Otorhinolaryngology.

[CR37] O’Leary UM, Rusch KM, Guastello SJ (1991). Estimating age-stratified WAIS-R IQS from scores on the Raven’s Standard Progressive Matrices. Journal of Clinical Psychology.

[CR38] Pellicano E, Gibson L, Maybery M, Durkin K, Badcock DR (2005). Abnormal global processing along the dorsal visual pathway in autism: A possible mechanism for weak visuospatial coherence?. Neuropsychologia.

[CR39] Poirel N, Mellet E, Houdé O, Pineau A (2008). First came the trees, then the forest: Developmental changes during childhood in the processing of visual local-global patterns according to the meaningfulness of the stimuli. Developmental Psychology.

[CR40] Pruneti CA, Fenu A, Freschi G, Rota S, Cocci D, Marchionni M (1996). Aggiornamento della standardizzazione italiana del test delle Matrici Progressive Colorate di Raven (CPM). Bollettino Di Psicologia Applicata.

[CR41] Raven J, Court JH, Raven JC (1998). Raven manual, section 1 (general overview) and section 2 (Coloured progressive matrices).

[CR42] Ropar D, Mitchell P (2001). Susceptibility to illusions and performance on visuospatial tasks in individuals with autism. Journal of Child Psychology and Psychiatry.

[CR43] Rutter M, Le Couteur A, Lord C (2003). The autism diagnostic interview—Revised (ADI-R).

[CR54] Shah A, Frith U (1983). An islet of ability in autistic children: A research note. Journal of Child Psychology and Psychiatry.

[CR44] Venuti P, Senese VP (2007). Un questionario di autovalutazione degli stili parentali: Uno studio su un campione italiano.

[CR47] White S, Saldaña D (2011). Performance of children with autism on the Embedded Figures Test: A closer look at a popular task. Journal of Autism and Developmental Disorders.

[CR48] Witkin HA (1950). Individual differences in ease of perception of embedded figures. Journal of Personality.

[CR49] Witkin HA, Goodenough DR, Karp SA (1967). Stability of cognitive style from childhood to young adulthood. Journal of Personality and Social Psychology.

[CR50] Witkin H, Oltman P, Raskin E, Karp S (1971). A manual for the embedded figures test.

[CR51] Zappullo I, Conson M, Zoccolotti P, Trojano L, Senese VP (2020). “Building blocks and drawing figures is not the same”: Neuropsychological bases of block design and Rey figure drawing in typically developing children. Child Neuropsychology.

[CR52] Zoccolotti P, Pizzamiglio L (1982). Measuring visual disembedding in a tachistoscopic presentation. Perceptual and Motor Skills.

